# Influence of Fumed Nanosilica on Ballistic Performance of UHPCs

**DOI:** 10.3390/ma16062151

**Published:** 2023-03-07

**Authors:** David Markusík, Luboš Bocian, Radoslav Novotný, Jakub Palovčík, Markéta Hrbáčová

**Affiliations:** Faculty of Chemistry, Brno University of Technology, Purkyňova 464/118, 612 00 Brno, Czech Republic

**Keywords:** ultra-high performance cement composite, differential efficiency factor, ballistic resistance, nanosilica

## Abstract

This research delves into the potential use of fumed nanosilica in ultra-high performance concrete for ballistic protection. First, the mechanical properties, slump flow, and specific gravity of UHPC with different contents of Aerosil 200 were determined. Then, calorimetric studies were conducted on these cement composites. Lastly, the differential efficiency factor and spalling area of UHPC with fumed nanosilica were determined. It was found out that the slump flow, the mechanical properties, and differential efficiency factor are slightly decreased by the addition of fumed nanosilica. However, the addition of the fumed nanosilica is beneficial in terms of the spalling area decrease and it is highly reactive during the induction period. Some of the results are supported by BSEM imaging.

## 1. Introduction

The high compressive strength, high durability, and dense matrix of ultra-high performance concrete is achieved by the optimization of packing density, use of very fine particulates, superplasticizers, and a low w/b ratio generally below 0.3. The use of steel fibers with different sizes and shapes makes the UHPC achieve ductility and high bending strength. However, slump flow is decreased by their incorporation, which can have an impact on the mechanical properties. These properties can be further improved by the use of silica fume (SF), which is very beneficial for the fiber–matrix interfacial bond and significantly decreases the CH content, which results in an increase in the mechanical properties [1,2,3,4,5,6].

Perhaps the most widely used pozzolanic nanomaterial in concrete is nanosilica (NS). It acts as a nucleation site for the CSH gel, which then fills out the pores. This leads to a higher packing density of UHPC. However, problems with uniform dispersion are a thing with the incorporation of NS into cementitious composites. Complex studies regarding NS in concrete have to be carried out in the future partly due to the several types of NS, among which colloidal nanosilica (CNS) and fumed nanosilica (FNS) are probably the most common types. At the same time, there are no available data on the ballistic performance of UHPC with NS at all [7,8,9,10].

There are some studies on the effects of CNS on properties of cement-based materials. Hou et al. studied the influence of CNS on cement hydration. They found that the hydration was accelerated by the addition of, in the early age, by accelerating the dissolution of cement particles and precipitation of products; however, it hindered hydration at later stages. Björnström et al. studied the hydration kinetics of C_3_S in the presence of CNS. They also came to conclusions similar to those of Hou et al. regarding the hydration, but the hindrance of hydration at later stages was not observed. Kontoleontos et al. studied the influence of CNS addition on the hydration of ultrafine cement. Immediate gain in mechanical properties was not observed, but the gain in compressive strength after 28 days was quite significant. On the other hand, a decrease in setting time was observed [11,12,13].

Ghafari et al. studied the influence of CNS addition on UHPC properties. Much more consumption of Ca(OH)_2_ was observed compared to the SF. A benefit in terms of compressive strength after 28 days was also observed. They also noticed a decline of slump flow with the addition of CNS. It was also observed that the CNS increased the bond strength between the aggregate and the binder. Chen et al. studied the influence of olivine CNS addition on UHPC properties. An increase in early age compressive strength was observed. Researchers Li et al. came to similar conclusions as previous researchers. Said et al. studied the influence of CNS on the properties of cement pastes and mortars. The consumption of Ca(OH)_2_ by CNS was so high that higher CNS additions above 3% acted only as a filler. Similar research with conclusions similar to those stated above was conducted by Senff et al. and Zapata et al. Aly et al. studied the influence of CNS on the properties of glass cement mortar. It was found out that the addition of CNS was beneficial for the increase in terms of fracture energy and impact strength [8,14,15,16,17,18,19].

Studies on the influence of FNS addition on cement-based materials are limited because they do not specifically classify the type of NS. This leads to the assumption that when the CNS or NS slurry is not stated in those articles, the researchers used the FNS. Jalal et al. studied the influence of combining SF and FNS on the properties of high-performance concrete (HPC). The authors mention that the addition of FNS reduces the risk of bleeding and segregation. It was also found that combining SF and FNS effectively increases mechanical properties. Givi et al. studied the influence of the addition of FNS of different sizes on the mechanical properties of cement/FNS blends. It was concluded that the smaller the diameter of FNS particles, the better the improvement of early age mechanical properties and vice versa. Tao studied the influence of addition of FNS in concrete to its water permeability and structure. The occurrence of denser CSH gel was observed. Similar study was conducted by Gaitero et al., but their study is interesting because they compared both the FNS and CNS as additions in cement pastes. It was concluded that the addition of CNS is more beneficial in terms of calcium leaching [19,20,21,22,23].

Another study by Kong et al. was one in which they studied the influence of agglomeration of FNS on the properties of cement-based materials. It was found out that both agglomerated and non-agglomerated FNS improved the microstructure, but the gel from agglomerated FNS could not work as a binder and that even two types of gels could be observed by electron microscopy. This, in turn, could mean that agglomerated FNS does not have seeding activity for CSH gel growth. Zyganitidis et al. studied the influence of the addition of FNS on the nanomechanical properties of cement pastes. A decrease in the nanomechanical properties due to the cement pastes not having enough time to hydrate was observed. Shekari et al. studied the influence of FNS addition on the properties of HPC. It was mentioned that the addition of FNS resulted in an increase of mechanical properties and a lower water absorption of the samples. Gesoglu et al. compared the effect of the addition of SF and FNS on the properties of UHPC. It was concluded that the addition of FNS is similar to that of SF, but the addition of NS is ten times more efficient in terms of the increase in packing density; however, it was also stated that the combined use of SF and FNS is definitely more beneficial than using them alone. Ghafari et al. studied the influence of FNS addition on UHPC properties. They presented results similar to some studies stated above on reactivity with Ca(OH)_2_, mechanical properties and slump flow [24,25,26,27,28].

It is believed that the first researchers to observe the synergistic effect between steel fibers and SF were Ramadoss et al. This synergistic effect is believed to be the result of the enhancement of the SF addition on the bond strength between steel fibers and matrix by eliminating the amount of Ca(OH)_2_ in the interfacial zone. The SF also has a filling effect on the UHPC matrix and strengthens the UHPC overall. The filling effect of the SF can be observed with the increase of specific gravity with its addition. One could assume that similar results regarding bond strength could be applied to NS and indeed, the enhancement of bond strength between steel fiber and NS was observed by Pi et al. One can also assume that if the filling effect of the NS was significant, it would also be observable by the increase in the specific gravity. It is worth to note that the bond strength between steel fiber and matrix is thought to be crucial for the impact resistance of UHPC [10,29,30,31,32].

The impact resistance can also be generally increased by the increase in the volume of steel fibers incorporated in the matrix. However, there are problems such as fiber balling or increased cost that come with increasing of the volume of the steel fibers in the matrix. That means that the one way of increasing the ballistic resistance of UHPC could be the addition of NS, and increasing the ballistic resistance by strengthening the bond between the steel fiber and UHPC matrix. This increase in ballistic resistance would then be attributed to the increase in mechanical properties since it is generally accepted that the increase in properties such as compressive and bending strength also increases the ballistic resistance of UHPC. As was stated before, there has been no published research at all on the ballistic performance of UHPCs with the addition of FNS, even though there is very little literature regarding the dynamic strength of UHPC with the addition of nanoparticles. On the other hand, there is a lot of research available on the ballistic performance or impact performance of UHPCs with different types of fibers or different types of aggregates. Thus, it could mean that it should be possible to compare the ballistic performance of UHPC with the addition of FNS to, say, UHPC reinforced with hybrid fibers [10,33,34,35,36,37,38,39,40,41,42,43,44,45,46].

**Figure 1:**
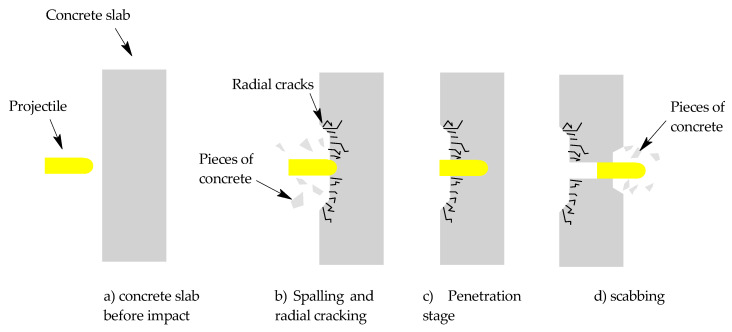
Basics of concrete projectile impact [10,47,48].

The basics of response of every concrete in regard to projectile impact: Firstly, there is the projectile impact after which the spalling occurs. The spalling phenomenon creates the entrance crater. After spalling, there is a phase known as tunneling, which consists of the projectiles going through material without any cratering. Lastly, there is phenomenon of scabbing associated with the projectile leaving the concrete. There is also compressive wave and radial cracking associated with the projectile impact on concrete along with possible deformation of the concrete slab. This can lead to the conclusions that the ballistic resistance of UHPC can be increased by the increase in the materials resistance to spalling, scabbing, and cracking. The basics of the ballistic response of concrete can be seen in the Figure 1 [10,47,48].

In this paper, the mechanical properties, slump flow, and specific gravity of UHPC with different amounts of Aerosil 200 FNS are evaluated. There is also isothermal calorimetry testing of composites provided to evaluate the effect of FNS on the matrix in terms of the hydration kinetics and, thus, provide a more complex description of the role of FNS in UHPC. Lastly, the depth of penetration (DOP) test is conducted on the UHPC with the addition of Aerosil 200 to calculate the value of differential efficiency factor (DEF), and the average spalling area is also evaluated. Some of the results are supported by BSEM imaging. It should be noted that we do not only present a set of never-before-published data in terms of ballistic resistance, we also degas each fabricated composite, which is rarely used in literature on UHPC and never used in case of FNS addition to UHPC [49]. At the same time, there is a clear lack of results on the influence of FNS addition on the UHPC bending strength of UHPC. It is believed by the authors that the importance of this research lies in the fact that it is a crucial piece of data for the understanding of the role and practical usability of nanomaterials in UHPCs. The purpose of this study was to find out if and how FNS affects the ballistic properties of UHPC.

## 2. Materials and Methods

### 2.1. Fabrication of Composites

The composition of each mixture can be seen in Table 1. One could find the mixture similar to that used in [49], but with the main difference that K_2_SO_4_ was not used. This chemical was not used in this study because the presence of K_2_SO_4_ caused the mixture to be very difficult to work with after only a few minutes of mixing. The percentage of Aerosil 200 was calculated as a weight percent of the binder (cement and silica fume). It should be noted that the KHCOO was pre-synthesized by the precipitation of KOH (technical quality 99%, Fichema, Brno-Líšeň, Czech Republic) and Ca (HCOO)_2_ (technical quality 98%, CHEMlogistics, Pardubice, Czech Republic).

Fine sand, cement, and SF were weighted into one bowl. Micronized sands and steel fibers were weighted in separate bowls. The first 248 mL of water were poured into a 250 mL cylinder and a superplasticizer was dosed into this cylinder with a 20 mL syringe. Then, potassium formate was added to the calinder and everything was mixed by turning the cylinder. The mixing was performed in a Hobart-type planetary mixer. First, the bowl with fine sand, SF, and cement were mixed at the slowest setting for 1 min to properly homogenize the mixture. The mixture of water, superplasticizer, and potassium formate was added after the 1 min. The mix was then mixed until plastification, after which the micronized sands were added and the mixing was conducted on the fastest setting after the addition of micronized sands. The remainder of the water was then added after 5 min of mixing. Steel fibers were then added slowly after 8 min of mixing. The mixing was conducted after 10 min and the minicone slump-flow test was conducted. The mixture was added to the mini-cone and the slump flow was determined after 30 s of lifting the cone.

The degassing of the mixture in the vacuum chamber was performed after the fresh compound was mixed and the slump flow was determined. The mixture was poured into the vacuum chamber, which was then sealed and the vacuum pump was turned on. The mixture was then mixed with the agitator until the boiling of water was observed. The degassed mixture was then quickly poured into the moulds for the fabrication of 3 samples with dimensions of 4 × 4 × 16 cm. Three mixes (6 specimens from each mix) were made from each type of mixture; so, 3 slump flow values were obtained for each mixture. The pouring into the moulds was conducted in the same way as in [49], in a fashion that the fibers were oriented along the longest dimension of the specimens. After pouring the mixtures into the moulds, the moulds were vibrated for 30 s and left to harden for 24 h, after which all the samples were demoulded, and specimens unsubjected to the evaluation of mechanical properties were cured under water for 7 and 28 days.

### 2.2. The Evaluation of the Specific Gravity

First, the specimens for the testing of mechanical properties with dimensions of 4 × 4 × 16 cm were weighted before the testing of mechanical properties after 7 and 28 days. Then, the weight of the specimens was divided by the volume of specimens and specific gravity was calculated and averaged.

### 2.3. Testing of Mechanical Properties

The evaluation of mechanical properties was conducted after 24 h (demoulding), 7 days, and 28 days. Three specimens were tested every time. All the tests were carried out according to ČSN EN ISO 196-1. First, the bending strength was evaluated with Instron 5895 with a 250 kN load cell. The span of supports measured 100 mm and the preload speed was 3 mm/min until the force of 5 kN was reached. Then, the loading rate was 0.08 kN/s.

The compressive strength testing was carried out on the concrete testing machine DESTTEST 3310 with a 3 MN load cell from Czech brand BetonSystem. The loaded area was always 1600 mm^2^ and the loading rate was 2.4 kN/s. Both ends of the samples used for the bending strength were used for the determination of compressive strength.

### 2.4. Isothermal Calorimetry

Isothermal calorimetry was performed only on the UHPC matrix with the same proportions as in Table 1. First, 50 g of composite mixture was fabricated and 10 ± 0.005 g of this mixture was poured into 20 mL ampoules made from plastic. The reference material was water demineralized in an amount that corresponded to the thermal capacity of the composite. The testing was conducted on TAM AIR from TA Instruments.

### 2.5. BSEM Imaging of Fracture Surfaces

The SEM used for BSEM imaging was the Zeiss EVO LS 10. The voltage was 15 kV. The specimens for the fracture surfaces were made by cutting the specimens for testing of the mechanical properties and breaking them with pliers afterwards.

### 2.6. Fabrication of Composites for Testing of Ballistic Properties

The composite used for ballistic testing was the composite with 0.5% of Aerosil 200; because of the results regarding bending strength, the slump flow was still relatively tolerable along with the price compromise for 1 m^3^ of mixture. The proportions were the same as in Table 1, but the dosage of each ingredient was calculated for the fabrication of 5 cylinder-shaped specimens with a diameter of 15.6 cm and depth of 4 cm. The mixing of this composite was first conducted in a pan-type concrete mixer LBM-75 from the Czech brand BetonSystem. This was carried out in a similar way to the fabrication of composite specimens for the testing of mechanical properties. The composite was then shoveled into the drum-type mixer with a volume of 120 L for the degassing. The degassing was repeated until there was visible boiling of the water observed. The composite was then poured into the moulds for the fabrication of cylinder-shaped specimens and vibrated with a specially made vibration motor. The samples were then left to harden for 24 h and then placed under water for 28 days of curing. The reference composite for the depth of penetration test had the same composition as that of the reference composite in Table 1.

### 2.7. Depth of Penetration Test

**Figure 2:**
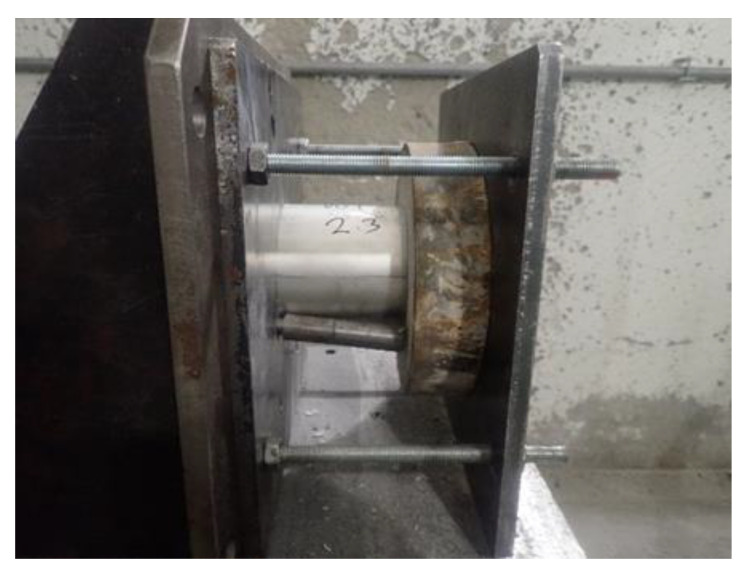
Cylindrical specimens of concrete with aluminum cylinders attached to their back side for DOP test.

The DOP test was conducted on cylindrical specimens with a diameter of 10 cm and length of 4 cm with a cylinder made of aluminum alloy with a diameter of 9 cm and length of 8 cm attached to each one on their back side. This can be seen in the detail in Figure 2. The aluminum alloy cylinders were made of AlCu_4_PbMg alloy with a density of 2.8085 g/cm^3^ according to the EN AW-2030-T4 standard. For this test, the 7.62 × 54 R cartridges (steel-core-armor-piercing incendiary projectile) with a mark velocity of 850 ± 20 m/s were used. The DOP test was performed with a universal ballistic breech with a 7.62 mm barrel and laser attached. The distance between the end of the barrel and the concrete slab was 10 m. The spacing area was determined as the average value of all of the 5 specimens used for the DOP test.

**Figure 3:**
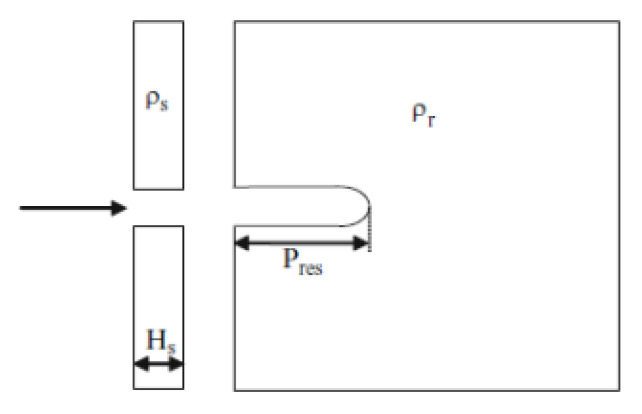
The dimensions used in the calculation of DEF.

The aluminum alloy cylinders were subjected to x-ray imaging after the test, and DOP was used for the calculation of the differential efficiency factor. The differential efficiency factor was calculated according to the equation

(1) $$DEF=\frac{\rho_{r}\left(P_{r}-P_{res}\right)}{\rho_{s}H_{s}},$$

where *ρ_r_* is the density of the aluminum alloy cylinder, *ρ_s_* is the density of material subjected to the DOP test, *P_r_* is the penetration depth in the aluminum alloy cylinder without the concrete cylinder, *P_res_* is the penetration into the aluminum alloy cylinder with the concrete cylinder, and *H_s_* is the length of the concrete cylinder. The dimensions used in the calculation of *DEF* in relation to the aluminum alloy cylinder can be seen in the scheme in Figure 3. Along the DEF, the spalling area was also determined.

## 3. Results and Discussion

### 3.1. Mechanical Properties, Slump Flow, and Specific Gravity

The results of the slump flow can be seen in Table 2. There is clearly no obvious decline in the slump flow with the lowest addition of Aerosil 200, but the slump flow steeply declines between the 0.25% addition of Aerosil 200. The decline in slump flow between the additions of 0.5% and 1.0% is not as steep as between the 0.25% and 0.5% addition. This agrees with the literature in the introduction and can be explained by the increased amount of water needed to sustain workability because of the higher specific surface area of the FNS compared to the SF. However, the trend between the data is not linear. This could perhaps be due to the difference in the number and size of the FNS agglomerates between the different dosage of FNS in mixtures. This could be a further direction to be explored in future research.

The average values of specific gravity of specimens after 7 and 28 days can be seen in Table 3 and Table 4. There seems to be almost no change in specific gravity of composites with Aerosil 200 compared to the reference composite. However, the composite with the addition of 0.5% of Aerosil 200 has the biggest specific gravity from all the composites after both 7 and 28 days; however, the change still is not enough to say that the addition of 0.5% of Aerosil 200 significantly increases the specific gravity. This could be attributed to the effects mentioned elsewhere together with the fact that the addition of only 0.5% of Aerosil 200 is not enough.

**Figure 4:**
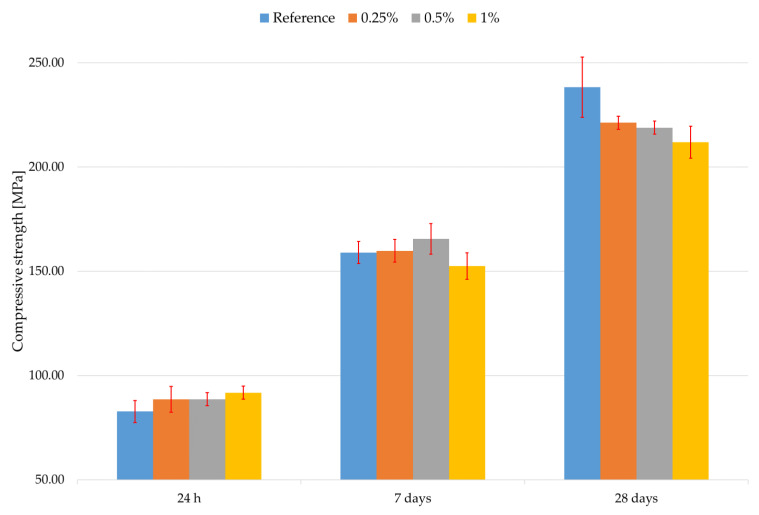
The compressive strength results.

The results of the evaluation of compressive strength evaluation can be seen in Figure 4. Error bars represent standard deviation. The percentage represents the addition of Aerosil 200 to the composite. It is clearly evident from the results in Figure 4 that there is no clear difference in compressive strength in the first 7 days of curing. There is also a clear decline in compressive strength after 28 days of curing with the addition of Aerosil 200. This is contrary to the literature revised above.

**Figure 5:**
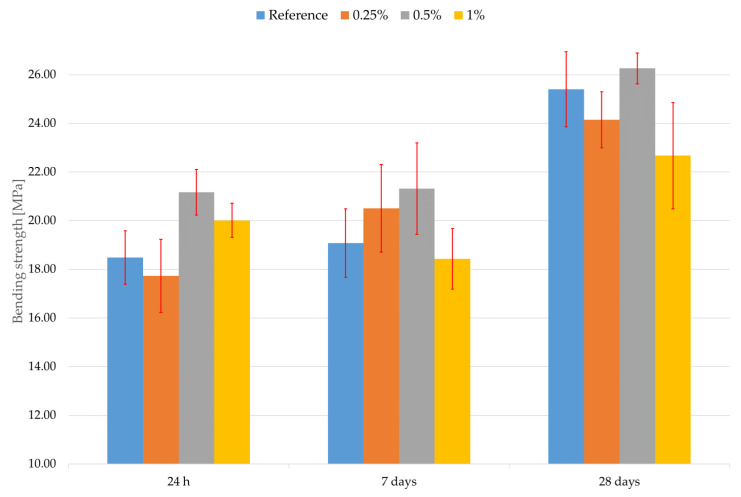
The bending strength results.

Similar results were observed in the case of bending strength, which can be seen in Figure 5. The error bars represent the standard deviation in Figure 5. The percentage represents the addition of Aerosil 200 to the composite. The discussion of results regarding the bending strength of UHPCs with the addition of FNS is quite difficult, because there is no literature containing these. The bending strength does not really seem significantly affected by the addition of Aerosil 200, similar to the compressive strength except for the composite with the addition of 0.5% Aerosil 200; however, there seems to be no justification for its usage for the increase in the bending strength after 28 days. Nevertheless, some addition of the FNS could be beneficial in terms of early-age strength when higher than the reference bending strength is needed. However, this comes with the disadvantage of a slightly lower compressive strength after 28 days and a higher cost of the composite.

As for the overall mechanical performance of the composites with the addition of Aerosil 200, it seems that proper homogenization of FNS in dry form could be a problem that is supported by the results of compressive strength. Recall that the authors of this research did not employ any specific method for the proper homogenization of FNS. However, that assumption is only partly supported by the bending strength results. It is worth noting that there is already a relatively high amount of SF present in the composites. This could mean that Aerosil 200 could act only as a filler because there would not be not enough Ca(OH)_2_ to react with FNS. This, however, seems to be relatively improbable because of the fast-reacting nature of the FNS, which will be supported by calorimetry data below. The only possible explanation in terms of this strand of thought is that the FNS agglomerates could react only at their surface; so, the unreacted inner parts of agglomerates would serve as a filler. This would then mean that the inner part of the agglomerate could not efficiently act as a filler because the agglomerates are held together mainly because of Van der Waals forces. The other possible way of thinking could go the other way round and suppose that there is not enough Ca(OH)_2_ to react with SF at relatively later stages. The SF would then act in the same way as agglomerated and unreacted FNS. Another possible situation is that the CSH gel that originated from the reaction of Ca(OH)_2_ and FNS slows the diffusion of water and ions between other parts of the mixture, thus slowing hydration down. This seems supported by the literature review, and it would mean that the main effect of FNS addition would be visible during the later ages (maybe even longer than 90 days). All the assumptions above need more research.

### 3.2. Isothermal Calorimetry

**Figure 6:**
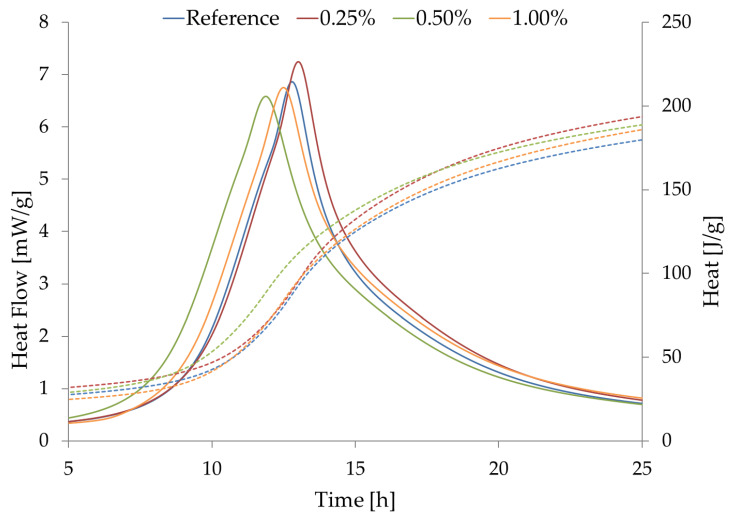
Isothermal calorimetry results.

The results of isothermal calorimetry can be seen in Figure 6. Both heat flow and heat curves can be seen in Figure 6. The setting acceleration can be clearly seen from the obtained data, but a major early-age acceleration of hydration is observed with Aerosil 200 that are higher than 0.25%. The major conclusion from the data presented in Figure 6 is, however, that FNS reacts and dissolves itself already in the induction period of setting. This is something that is not often stated in the revised above.

### 3.3. BSEM Imaging

**Figure 7:**
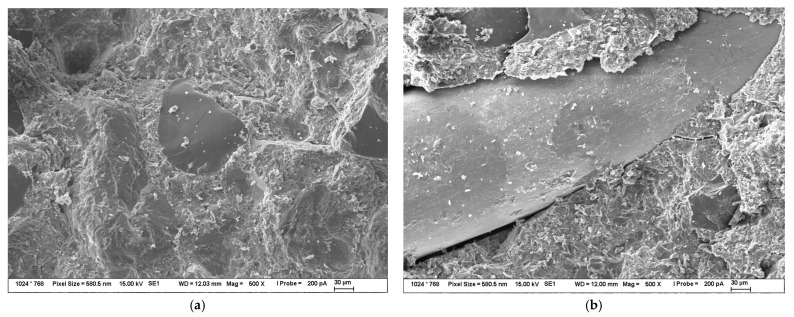
BSEM images of fracture surfaces of reference composite (**a**) fracture surface of composite without steel fiber; (**b**) fracture surface of composite with steel fiber.

BSEM images of the fracture surfaces of the reference composite can be seen in Figure 7. The bigger visible particles in Figure 7 are the particles of dust, etc. that stuck to the surface after the breaking of the composite with pliers. The fracture surface of the micronized sand can be clearly seen in the middle of Figure 7a, which is evidence of good adhesion between the binder and fillers even without the addition of Aerosil 200. However, the readers can clearly see that despite there being some matrix visible on the steel fiber in Figure 7b, the amount of matrix sticking to the fiber is quite limited.

**Figure 8:**
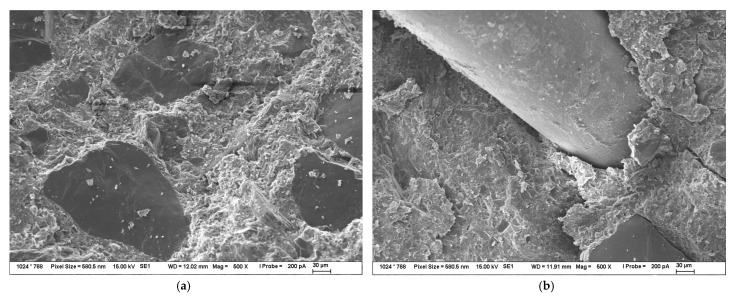
BSEM images of fracture surfaces of composite with the addition of 0.5% of Aerosil 200 (**a**) fracture surface of composite without steel fiber; (**b**) fracture surface of composite with steel fiber.

BSEM images of composite fracture surfaces with the addition of 0.5% of Aerosil 200 can be seen in Figure 8. The bigger visible particles in Figure 8 are the particles of dust, etc. that stuck to the surface after the breaking of the composite with pliers. The BSEM image of the fracture surfaces with micronized sand in Figure 8a is almost in same as the Figure 7a. More interesting is Figure 8b, in which it is clearly visible that there is much more matrix sticking to the fiber surface, which leads to the conclusion that the addition of FNS leads to the enhancement of the bond strength between the steel fiber and the UHPC matrix.

### 3.4. Differential Efficiency Factor and Average Spalling Area Results

The results of the calculated differential efficiency factor and the average spalling area with standard deviation are summarized in Table 5. It should be noted that six specimens were used for the ballistic testing of the reference composite because there was one failure to hit the specimen. Images from which all the data can be gathered can be seen in Appendix A. We can see from the data available in Table 5 that the DEF of composite with the addition of 0.5% of Aerosil 200 is slightly lower than that of the reference composite. The standard deviation is also higher than in the case of the reference composite. There is, however, a much lower average spalling area along with standard deviation in the case of composite with the addition of 0.5% of Aerosil 200. This means that even though the overall ballistic resistance of such a composite is lowered, the composite should provide more ballistic protection in terms of hits that land near themselves. This could in turn mean that the composite with the addition of FNS provides more durability in terms of ballistic protection. This is probably due to the increased bond strength between the UHPC matrix and the steel fibers. However, increasing the bond strength can, however, make the UHPC more brittle; thus, decreasing the average value of DEF.

It can be said though that the addition of FNS into the UHPC is not anywhere near as effective in increasing the ballistic resistance of UHPC as replacing part of the silica dans with aggregate such as corundum or basalt [42]. It is also not nearly as beneficial as using steel and PP fiber mixtures, which really beats the use of FNS for ballistic purposes because of the cheaper nature of the PP fibers compared to FNS [35].

## 4. Conclusions

The conclusions can be listed as follows:

1. Slump flow is negatively affected by the addition of FNS, but the relation between the size, number of agglomerates, and the homogenization of mixture on the slump flow of UHPC should be the subject of future research. It was also found out that the addition of FNS did not lead to the significant increase in specific gravity, albeit the specific gravity of composites with the addition of 0.5% of the Aerosil 200 was always higher than specific gravity of any other composite.
2. The addition of FNS can affect mechanical properties both positively and negatively. The slightly reduced compressive strength in our case can be mainly attributed to the poor homogenization of the FNS in the mixture. There is also some concern regarding the kinetics of FNS hydration, but this has to be further investigated in the future. The addition of FNS can be slightly beneficial to the early-bending strength of UHPC, albeit it should be stated that it does not add almost anything in terms of bending strength at later ages.
3. It was also observed that the addition of FNS accelerates hydration at the early age and that it even begins to dissolve during the induction period. Both the heat flow and heat are enhanced.
4. BSEM imaging of the fracture surface revealed that the addition of FNS enhances the bond strength between the UHPC matrix and the steel fibers, but the bond strength between the aggregate and the UHPC matrix is not affected at all. The improved bond strength between steel fibers can be used to explain the ballistic performance of this composite. However, there is some concern about the brittle failure of the composite when the bond strength between the steel fiber and the UHPC matrix is enhanced.
5. The addition of FNS slightly decreases the average value of DEF and increases its standard deviation. On the other hand, the average value of the spalling area and the standard deviation are both lowered, which could as a result mean that the composite with FNS is more durable in terms of ballistic protection in terms of multiple near hits. However, it is not nearly as effective in increasing the ballistic resistance of UHPC as some other means presented in the literature.

## Figures and Tables

**Table 1:** Mixture compositions.

Ingredients	Reference	0.25% Aerosil 200	0.5% Aerosil 200	1.0% Aerosil 200
Fine sand according to ČSN EN 196-1 (Filtrační písky Chlum, Czech Republic)	1980 g	1980 g	1980 g	1980 g
Micronized sand ST-2 (Sklopísek Střeleč, Czech Republic)	135 g	135 g	135 g	135 g
Micro-dorsilit 110 (Dorfner, D)	405 g	405 g	405 g	405 g
CEM I 52.5 R-SR 5 white (Aalborg Portland, DE)	864 g	864 g	864 g	864 g
Silica fume RW Füller-Q (Elkem, D)	216 g	216 g	216 g	216 g
Steel fibers 12.5 × 0.2 mm (KrampeHarex, D)	300 g	300 g	300 g	300 g
Potassium formate (synthesized)	34.4 g	34.4 g	34.4 g	34.4 g
Superplasticizer MasterGlenium ACE 446 (BASF, D)	45 mL	45 mL	45 mL	45 mL
Aerosil 200 (Evonik, USA)	0 g	2.7 g	5.4 g	10.8 g
Demineralized water	278 mL	279 mL	280 mL	281 mL

**Table 2:** Slump flow results.

Mixture	Slump Flow [mm]
Reference	179
0.25% Aerosil 200	176
0.5% Aerosil 200	153
1.0% Aerosil 200	143

**Table 3:** The average values of specific gravity of specimens after 7 days.

Mixture	Specific Gravity [kg/m^3^]
Reference	2574
0.25% Aerosil 200	2567
0.5% Aerosil 200	2580
1.0% Aerosil 200	2550

**Table 4:** The average values of specific gravity of specimens after 28 days.

Mixture	Specific Gravity [kg/m^3^]
Reference	2540
0.25% Aerosil 200	2548
0.5% Aerosil 200	2606
1.0% Aerosil 200	2560

**Table 5:** Ballistic test results.

Composite	DEF [-]	Average Spalling Area [mm^2^]
Reference	0.878 ± 0.044	3600 ± 740
0.5% Aerosil 200	0.855 ± 0.056	2692 ± 462

**Figure A1:**
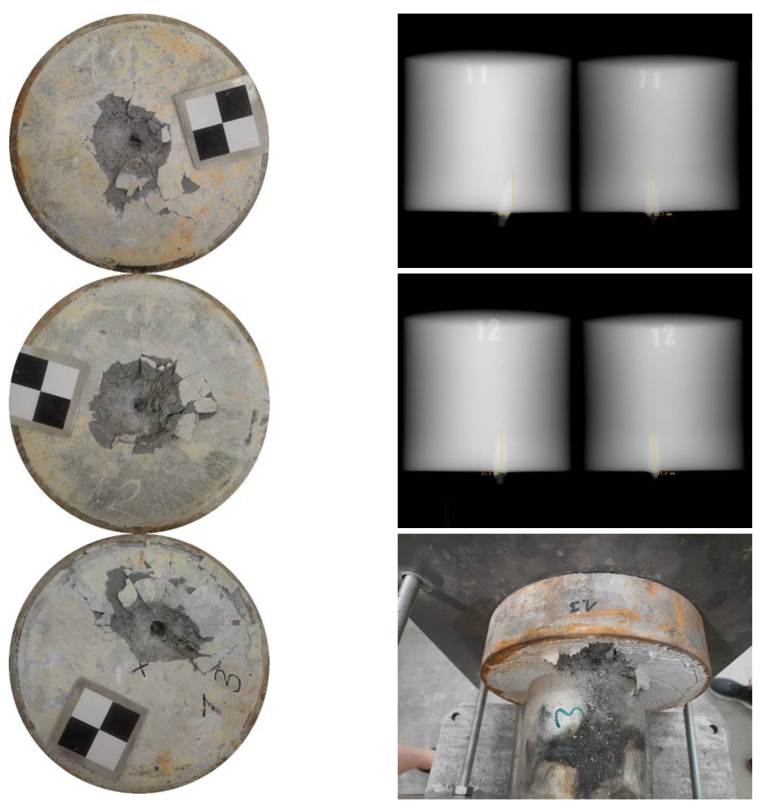
Set of data obtained from the DOP test of reference composite; left-data for obtaining the average spalling area for reference composite, right-data for calculating the DEF values.

**Figure A2:**
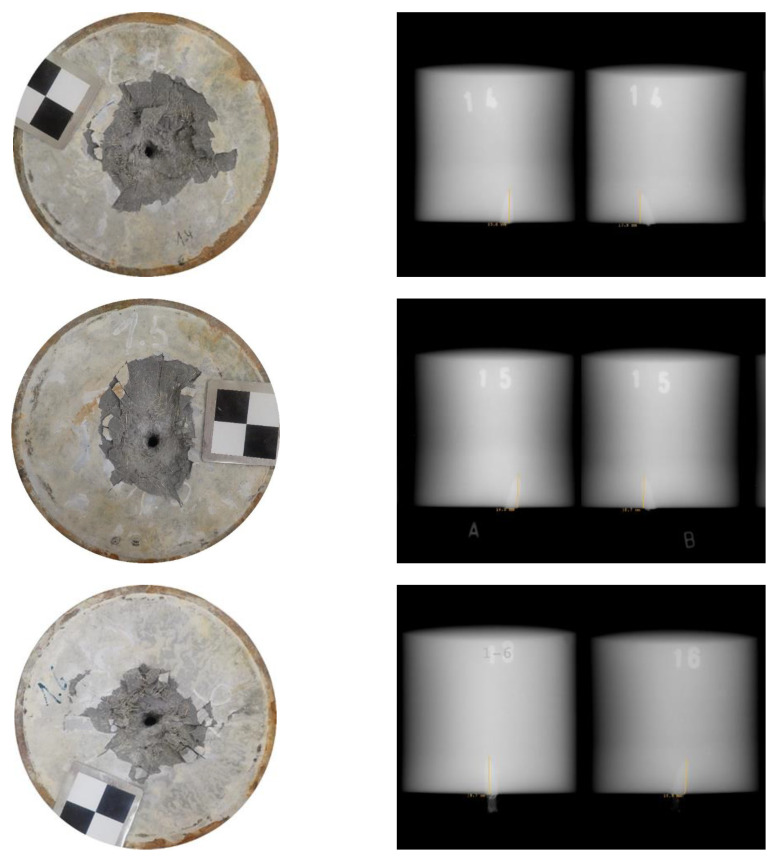
Another set of data obtained from the DOP test of reference composite; left-data for obtaining the average spalling area for reference composite, right-data for calculating the DEF values.

**Figure A3:**
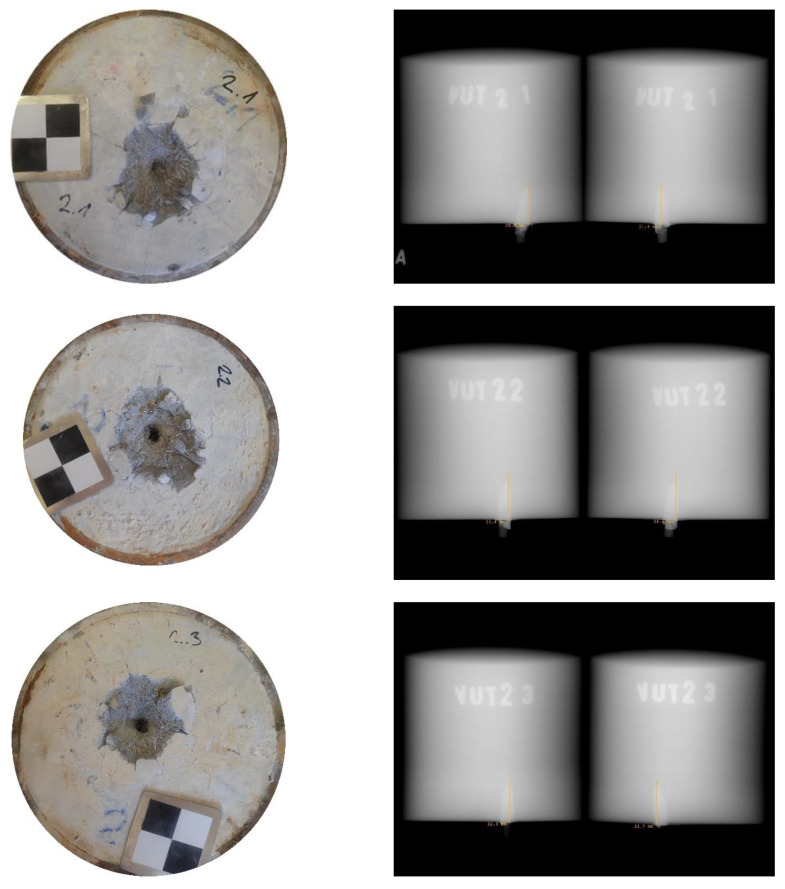
Set of data obtained from the DOP test of reference composite; left-data for obtaining the average spalling area for reference composite, right-data for calculating the DEF values.

**Figure A4:**
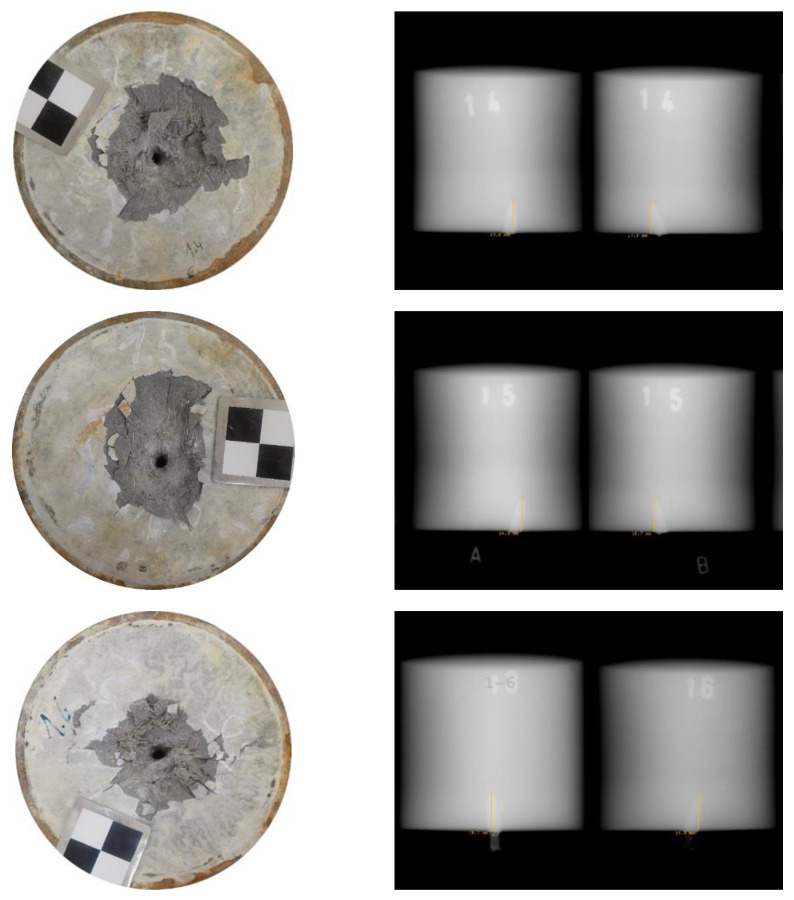
Set of data obtained from the DOP test of composite with the addition of 0.5% of Aerosil 200; left-data for obtaining the average spalling area for reference composite, right-data for calculating the DEF values.

**Figure A5:**
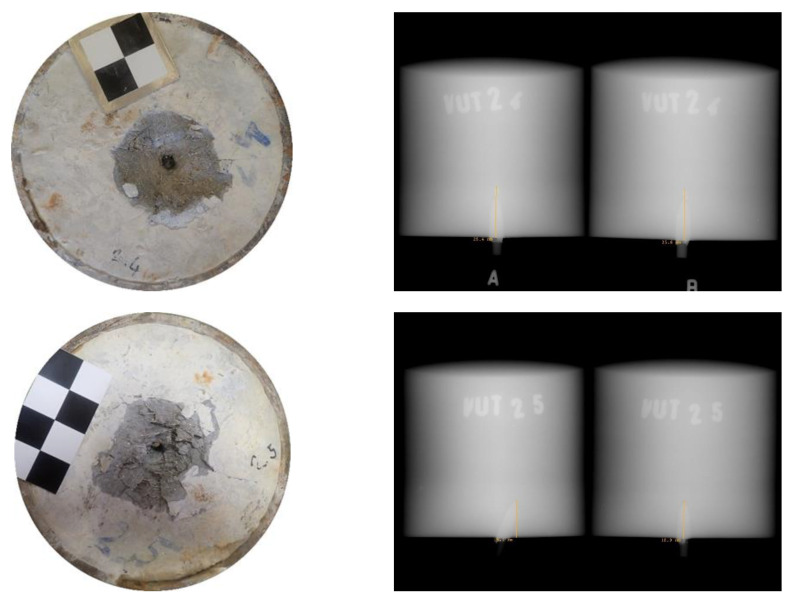
Another set of data obtained from the DOP test of composite with the addition of 0.5% of Aerosil 200; left-data for obtaining the average spalling area for reference composite, right-data for calculating the DEF values.

## Data Availability

Not applicable.
